# Severity of heterotopic ossification in patients following surgery for hip fracture: a retrospective observational study

**DOI:** 10.1186/s12891-019-2725-7

**Published:** 2019-07-27

**Authors:** Daichi Hayashi, Elaine S. Gould, Corey Ho, Dennis L. Caruana, David E. Komatsu, Jie Yang, Chencan Zhu, Musa Mufti, James Nicholson

**Affiliations:** 1 Department of Radiology, Stony Brook Medicine, HSC 4-120, Stony Brook, NY 11794 USA; 2 Department of Radiology, University of Colorado School of Medicine, Aurora, CO USA; 3 Yale School of Medicine, New Haven, CT USA; 4 Department of Orthopedics, Stony Brook Medicine, Stony Brook, NY USA; 5 Department of Family, Population and Preventive Medicine, Stony Brook University, Stony Brook, NY USA; 6 Department of Applied Mathematics and Statistics, Stony Brook University, Stony Brook, USA

**Keywords:** Hip, Arthroplasty, Postoperative complications, Orthopedic procedures, Radiography

## Abstract

**Background:**

Heterotopic ossification (HO) is a relatively common complication following hip surgery treated with open reduction and internal fixation, total arthroplasty or hemiarthroplasty. Development of HO after hip surgery is an important clinical issue as it can affect functional status. We aimed to determine whether there was association between severity of heterotopic ossification about the hip and the interval between the time of hip fracture and surgery.

**Materials and methods:**

Our retrospective study included 151 patients (age range 33–95 years) treated for hip fractures by hemiarthroplasty. Medical records were reviewed for time interval to surgery, laterality, surgical approach, and patient age. Patients who had any post-operative complications were excluded. Radiographs were semiquantitatively assessed for the degree of heterotopic ossification based on Brooker Classification (5-point scale). Statistical analysis was performed utilizing Chi-square, Kruskal-Wallis, and Score tests, and also a proportional odds model (significance level set at 0.05).

**Results:**

Thirty eight patients had no heterotopic ossification, 43 had class 1, 55 had class 2, and 15 had class 3 or greater heterotopic ossification. The majority of patients (59.6%) had surgery within 2 days of acute injury. Severe heterotopic ossification (HO 3+) was associated with the longer interval between the time of acute hip fracture and surgery (median 6 days) vs. median 2 days in all other groups (HO classes 0–2) (*p* = 0.0015). The odds ratio and 95% CI for one level higher HO class was 1.296 (1.152, 1.459), which meant that the odds of having HO class one level higher increased by about 29.6% for every one-day increase in the days to surgery. No significant association was found for other variables.

**Conclusion:**

Class 3 or greater HO was associated with longer time interval between time of acute hip fracture and surgery compared to all other groups (HO class 0–2).

## Background

Heterotopic ossification (HO) is a relatively common complication following hip surgery treated with open reduction and internal fixation (ORIF), total arthroplasty (THA) or hemiarthroplasty. For example, a recent study showed the prevalence of HO after minimally invasive short-stem THA using a modified anterolateral approach to be 7.8% (16 out of 216 cases) [1]. Development of HO after hip surgery is an important clinical issue as it can affect functional status [2]. Means to prevent or reduce HO have been explored and include pre-operative [3] and post-operative irradiation [4] and the use of non-steroidal anti-inflammatory drugs [5, 6]. Rate and degree of HO after THA were shown to be affected by patient gender [7], surgical approach [8, 9] as well as type of surgery [10]. Moreover, African-American ethnicity has been shown to be an independent risk factor for HO formation after THA [11]. An increased occurrence of HO was also reported specifically in patients with ankylosing spondylitis, elevated preoperative serum inflammatory markers and prolonged duration of surgery [12]. Previous hip HO formation and bilateral hypertrophic hip osteoarthritis are other known risk factors for HO [13].

HO shows progression over time in radiographic appearance. In early stage, it is typically a soft tissue mass without overt calcification and can often be missed. In the mineralization stage which can occur within 10 days after causative insult, calcification usually starts peripherally. Lesions can also be poorly organized without recognizable mineralization pattern. In mature HO, cortical bone is formed. The degree of HO can be semi-quantitatively assessed using the Brooker classification (grade 1–4) [14]. So far, the relationship between time of surgery and the severity of postoperative HO has not been well established in the literature. In a single center retrospective study, interval from injury to surgery was not statistically significantly associated with development and severity of HO in a cohort of 241 patients with acetabular fractures [15]. Another retrospective study showed that patients who underwent THA for acetabular fracture early after injury had higher (4-fold) chance of developing HO [16]. However, in other retrospective and prospective studies of HO after surgical repair of elbow fractures, longer time to surgery was an independent predictor of HO [17, 18]. Thus, the existing literature evidence on the relationship between time to surgery and the incidence/severity of HO remains controversial. We hypothesized that the longer the time to surgery after hip injury, the more severe the postoperative HO will become.

The aim of our study was to determine the association between severity of heterotopic ossification around the hip joint and the interval between the time of injury and surgery.

## Methods

### Subjects

Our retrospective study received Institutional Review Board approval and the need for informed consent from the patients was waived. We retrospectively reviewed the medical records in our institution for patients who had hip bipolar hemiarthroplasty (CPT codes 27125 and 27236) performed by an orthopedic surgeon to treat femoral neck fractures between 01/01/2003 and 11/22/2013. For each patient, the date of surgery, laterality (left or right hip), surgical approach (lateral, posterior, anterolateral), patient age, date of injury and interval between injury and surgery (days) were recorded. We excluded patients who had postoperative complications such as re-fracture, and hardware related complications including loosening, fracture and infection.

### Radiographic evaluation of heterotopic ossification

**Fig. 1:**
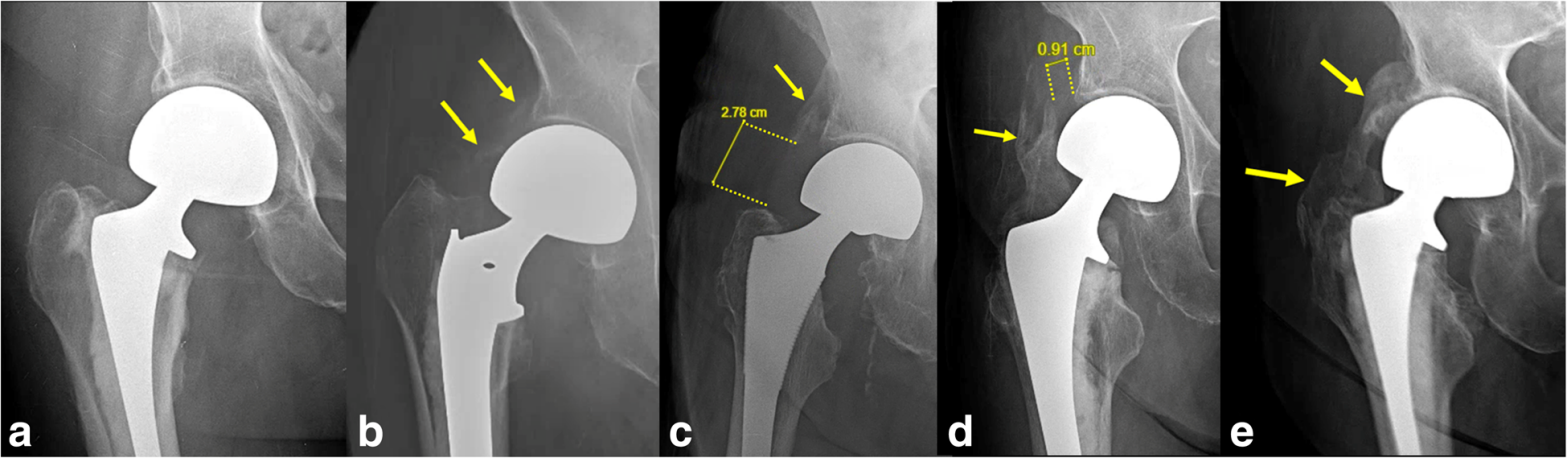
Brooker classification of heterotopic ossification in the hip. **a** Class 0 – no heterotopic ossification is noted about the hip; **b** class 1 – small islands of soft tissue ossifications are noted about the hip (arrows); **c** class 2- a moderate spur (arrow) arising from the right acetabulum, leaving an osseous gap of greater than 1 cm measured from the greater trochanter; **d** class 3 – a very large spur (arrow) arising from the greater trochanter of the right proximal femur approaching the acetabulum, leaving an osseous gap of less than 1 cm; **e** class 4 – arrows show large spurs arising from the acetabulum and also greater trochanter of the right proximal femur, resulting in ankylosis

Using post-operative radiographs of the pelvis/hip, the severity of post-operative heterotopic ossification was semi-quantitatively graded using the Brooker classification as follows: class 1 = islands of bone within the soft tissues about the hip; class 2 = bone spurs from the pelvis or proximal end of the femur, leaving at least one centimeter between opposing bone surfaces; class 3 = bone spurs from the pelvis or proximal end of the femur, reducing the space between opposing bone surfaces to less than one centimeter; class 4 = apparent bone ankyloses of the hip (Fig. 1 and Table 1) [14]. Pelvis/hip radiographs were read in consensus by one attending musculoskeletal radiologist and one musculoskeletal radiology fellow blinded to clinical information. If a patient had more than one follow-up radiographs, the most recent radiograph was reviewed.

**Table 1:** Brooker classification of heterotopic ossification in the hip

Class	Definition
1	Islands of bone within the soft tissues about the hip
2	Bone spurs from the pelvis or proximal end of the femur, leaving at least 1 cm between opposing bone surfaces
3	Bone spurs from the pelvis or proximal end of the femur, reducing the space between opposing bone surfaces to less than 1 cm
4	Apparent bone ankylosis of the hip

### Statistical analysis

Since the number of subjects with HO class “3” and “4” were low (*N* = 11 and 4, respectively), they were combined together as “3+” in the following analysis. Chi-square tests with exact *P*-values based on Monte Carlo simulation were utilized to examine the marginal association between categorical variables (*gender, approach, side*) and HO class. Kruskal-Wallis tests were used to examine the marginal association between continuous variables (*days to surgery, age*) and HO class. Proportional odds model was used to further quantify the relationship between HO class (treated as ordinal variable) and days to surgery. Score test was utilized to confirm the proportional odds assumption. Of note, these additional analyses were not performed for other variables, since the results of the aforementioned Chi-squared test and Kruskal-Wallis test showed statistically non-significant association with the severity of HO with *p*-values well above 0.05, and thus calculation of odds ratio would also be non-significant. Statistical analysis was performed using SAS 9.4 and significance level was set at 0.05 (SAS Institute Inc., Cary, NC).

### Evaluation of possible influence of time interval between the date of surgery and date of follow-up radiograph

As we reviewed the obtained dataset, it became clear that there was a large variation in time interval between the date of surgery and date of follow-up radiograph (range 76–3049 days). One might therefore think patients who had longer interval for follow-up were more likely to have HO. We therefore assessed the distribution of follow-up interval (in days) among different HO categories (0,1,2,3+) and assessed if the duration of time interval between the date of surgery and follow-up radiograph affected the severity of HO.

## Results

**Fig. 2:**
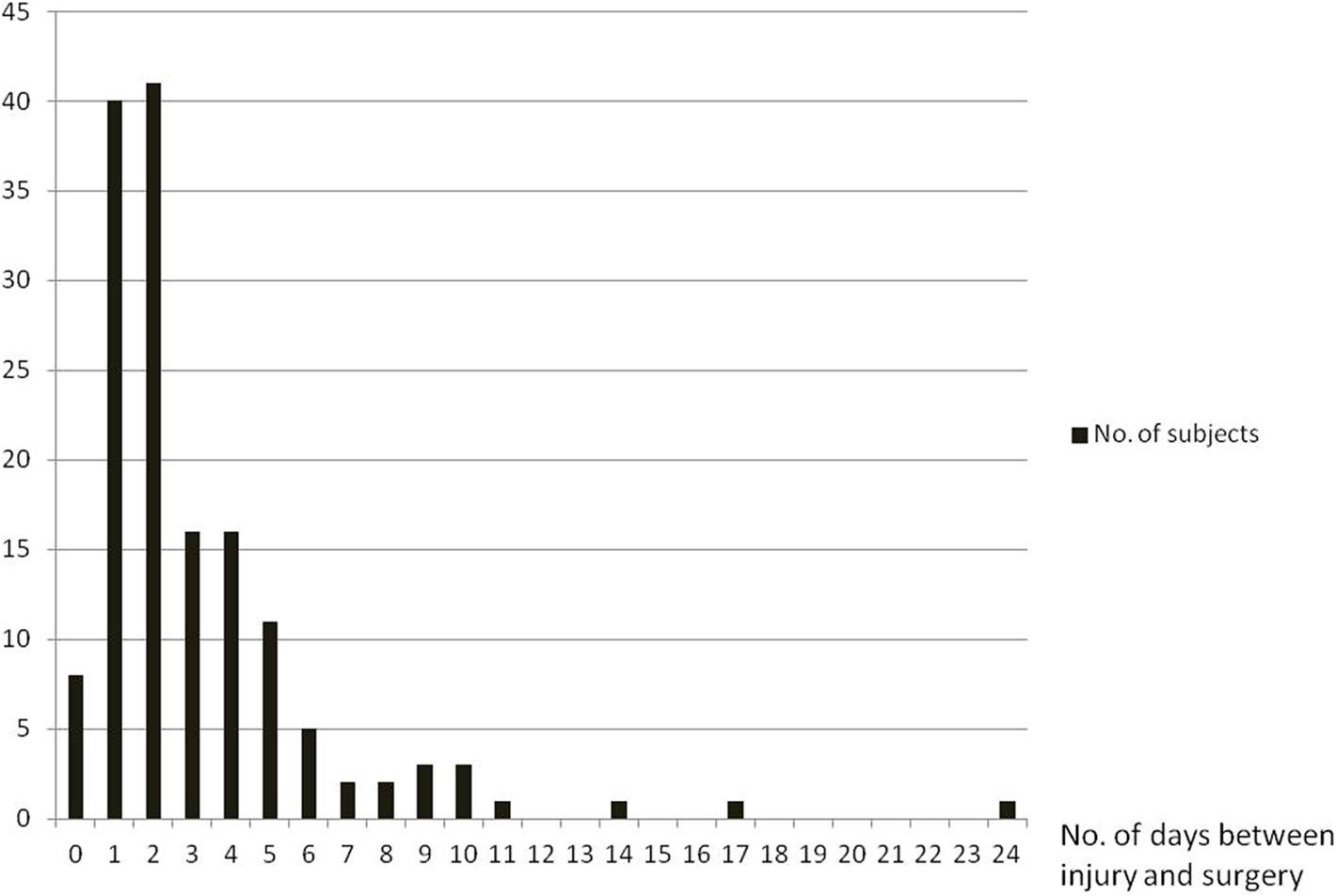
Distribution of subjects according to the number of days to surgery. A majority of patients (90 of 151, 59.6%) had a surgical intervention within 2 days of injury. Subjects who had extended delays of surgery all had mitigating medical reason which prevented medical clearance for undergoing surgery

Table 2 shows the descriptive table for patients’ gender, age, days to surgery, surgery approach and side by HO class. Most of the patients were 65 years or older, but 18 patients younger than 65 years received bipolar hemiarthroplasty due to clinical indications such as delayed surgery secondary to compromised systemic status, poor general health that would prevent a second operation or displaced fracture which was several days old. Severe heterotopic ossification (HO 3+) was associated with the longer interval between the time of acute hip fracture and surgery (median 6 days) vs. median 2 days in all other groups (HO classes 0–2) (*p* = 0.0015). In other words, HO class 1 and HO class 2 had the same the interval between the time of acute hip fracture and surgery as patients without ossifications. Patient age did not significantly differ amongst different HO classes with a large range of overlap around the age 80 (*p* = 0.2812). Patient gender was also not associated with HO class (*p* = 0.0705) although higher proportion of male patients (compared to female patients) had HO class 2 (44.74% vs. 33.63%) and class 3+ (15.70% vs. 7.96%). Conversely, a higher proportion of female patients had HO class zero (30.09% vs. 10.53%) compared to men. Surgical approach and side of surgery showed essentially no association with HO class, with *p*-values much higher than 0.05 (*p* = 0.1882 for surgical approach, *p* = 0.7383 for side of surgery). Figure 2 shows the distribution of subjects according to the number of days to surgery. A majority of patients (90 of 151, 59.6%) had a surgical intervention within 2 days of presentation.

**Table 2:** Descriptive table for patients’ characteristics and surgery information by HO class

Variables		Total (*N* = 151)	HO class 0 (*N* = 38)	HO class 1 (*N* = 43)	HO class 2 (*N* = 55)	HO class 3+ (N = 15)	*P*-values
Days to surgery		2 ± 3	2 ± 1	2 ± 3	2 ± 3	6 ± 6	0.0015
Age		81 ± 11	82.5 ± 16	83 ± 11	80 ± 11	77 ± 10	0.2812
Gender	Female	113 (74.83%)	34 (30.09%)	32 (28.32%)	38 (33.63%)	9 (7.96%)	0.0705
	Male	38 (25.17%)	4 (10.53%)	11 (28.95%)	17 (44.74%)	6 (15.79%)	
Surgical Approach	Anterolateral	4 (2.65%)	0 (0.00%)	3 (75.00%)	1 (25.00%)	0 (0.00%)	0.1882
	Lateral	33 (21.85%)	5 (15.15%)	8 (24.24%)	15 (45.45%)	5 (15.15%)	
	Posterior	114 (75.50%)	33 (28.95%)	32 (28.07%)	39 (34.21%)	10 (8.77%)	
Side of surgery	Left	82 (54.30%)	20 (24.39%)	24 (29.27%)	28 (34.15%)	10 (12.20%)	0.7383
	Right	69 (45.70%)	18 (26.09%)	19 (27.54%)	27 (39.13%)	5 (7.25%)	

*For categorical variables, *p*-value was based on Chi-squared test with exact *p*-value from Monte Carlo simulation; for continuous variables, median +/− interquartile range were reported and *p*-value was based on Kruskal-Wallis test

Proportional odds model was used to further analyze the relationship between HO class and time to surgery. The odds ratio and 95% CI for one level higher HO class was 1.296 (1.152, 1.459), which means that the odds of having HO class one level higher increases by about 29.6% for every one-day increase in the days to surgery. This meant that as days to surgery increased, patients were more likely to have higher class of heterotopic ossification.

**Fig. 3:**
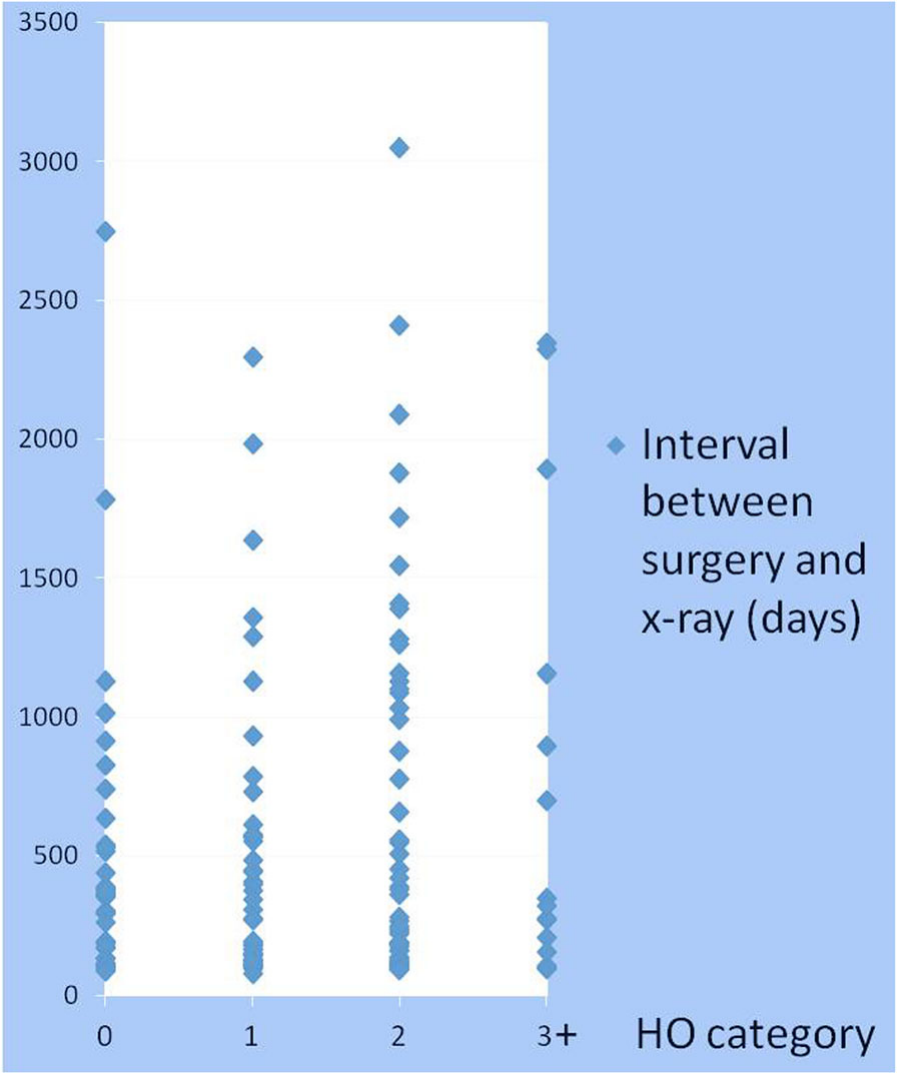
Distribution of follow-up interval among different HO categories. The median follow-up interval for class 0 was 353.5 days (range, 89–2746); class 1 was 306 days (range, 76–2296); class 2 was 279 days (range, 94–3049); and class 3+ was 321 days (range, 95–2345). Thus, shortest median follow-up interval was observed with class 2 HO group, and the median follow-up interval was actually shorter for class 3+ group compared to class 0 group

The median number of days between the surgery and follow-up radiograph was 321, with a range of 76–3049. In our study sample, distribution of follow-up interval among different HO categories (0,1,2,3+) was similar (Fig. 3) and longer follow-up interval did not necessarily correspond to higher HO category. The patient with longest follow-up interval (3049 days) had class 2 HO, and there was a patient who had no HO at 2746 days. Conversely, a patient whose follow-up radiograph was taken at 96 days had class 4 HO. When our sample was stratified according to HO class, the median follow-up interval for class 0 was 353.5 days (range, 89–2746); class 1 was 306 days (range, 76–2296); class 2 was 279 days (range, 94–3049); and class 3+ was 321 days (range, 95–2345). Thus, shortest median follow-up interval was observed with class 2 HO group, and the median follow-up interval was actually shorter for class 3+ group compared to class 0 group.

## Discussion

Our results noted a strong association between the severity of postoperative HO and time to surgery. Available literature evidence regarding risk of postoperative HO and its association with time interval between injury and surgery has been mixed. No significant association between development/severity of HO and time interval from injury to surgery was found in d’Heurle et al’s study in which hip fracture patients were evaluated [15], while Chemaly et al. reported patients who underwent ‘early’ surgery (total hip arthroplasty within 60 days of injury) had higher incidence of HO compared to those who underwent ‘late’ surgery (more than 60 days after injury) [16]. Hong et al. showed that the risk of developing HO after elbow fracture and surgical fixation increased with time to surgery: using the ≤24 h category as the reference, the 2–7 days category had an OR of 3.78 (95%CI, 1.12–12.78; *P* = 0.033) and the > 7 days category an OR of 10.62 (95%CI, 2.96–38.09; *P* = 0.001) [17]. Also, Bauer et al. reported longer time to surgery was a risk factor for the development of HO, with subjects waiting 8 or more days for surgery having 12 times the odds of HO than subjects having surgery within a day of injury [18].These last two studies are in line with the findings of our study. However, exact reason for this finding is yet to be determined, and given mixed literature evidence, it remains to be a controversial issue.

Patient age can be an important factor for severity of HO, as one might consider younger patients to have more tendency for bone formation compared to elderly patients. However, our analysis showed that age is not a statistically significant factor associated with severity of HO. Each HO grade had very similar median age and IQR, as well as age range (grade 0, min 54 years and max 94 years; grade 1, min 52 years and max 95 years; grade 2, min 34 years and max 94 years; grade 39, min 33 years and max 86 years). Upon detailed review of individual patient data, there were three particularly young patients. The patient aged 33 years had grade 4 HO, in whom interval between surgery and follow-up radiograph was 1894 days. The patient aged 34 years had grade 2 HO with follow-up interval of 2410 days. The patient aged 37 years had grade 4 HO with follow-up interval of 1159 days. Then, the next youngest patient was aged 50 years, who had grade 2 HO. Thus, there were only three ‘outliers’ in terms of age distribution of our study sample, and two of these three subjects had grade 4 HO. This could be related to speculation that young patients may be more likely to get severe HO. However, the number of patients is too small to derive statistically meaningful conclusion regarding these very young patients. For patients aged 50–69 years, there were only grade 0, 1 and 2 HO’s. The remainder of high grade HO’s (class 3 and 4) were only found in patients aged 70 years or older. The oldest age for grade 3 HO was 91 years and that for grade 4 HO was 86 years. Despite all these detailed observations, overall age does not seem to be significant confounding factor for HO grade severity, as demonstrated by our formal statistical analysis.

We did not adjust the analysis for any demographic factors which were previously reported as possible risk factors (such as gender, surgical approach, ethnicity, etc.), as our samples did not suggest that these were risk factors. Nonetheless, we fit a model that adjusted for gender and surgical approach. The results suggest that after further adjusting for gender and surgical approach, the odds of having HO class one level higher increases by about 30.2% for every one-day increase in the days to surgery (OR = 1.302, 95% CI: 1.158–1.463, *p* < 0.0001.) This is in line with our original findings.

A limitation of our study is that there was a large variation in time interval between the date of surgery and date of follow-up radiograph (range 76–3049 days). However, in our study sample, distribution of follow-up interval among different HO categories (0,1,2,3+) was similar (Fig. 3). Of note, there were total 50 patients who had radiographic follow-up within 180 days of surgery. Of these patients, 11 patients (22%) had class 0 HO, 17 patients (34%) had class 1 HO, 18 patients (36%) had class 2 HO, 4 patients (8%) had class 3+ HO. In contrast, 101 patients had follow-up x-ray longer than 180 days after surgery. Of these patients, 27 patients (27%) had class 0 HO, 26 patients (26%) had class 1 HO, 37 patients (37%) had class 2 HO, 11 patients (10%) had class 3+ HO. Fisher’s exact test shows the distribution of HO class is not significantly different between early and late follow-up groups (*p*-value = 0.7323). Moreover, when only using 101 patients who had follow-up x-ray longer than 180 days after surgery, the estimated odds ratio for days to surgery was 1.354 with 95% CI: 1.159–1.583 (*p* = 0.0001) or was 1.374 with 95% CI: 1.176–1.606 (*p* < 0.0001) after further adjusting for gender and surgical approach, which suggested that the odds of having HO class one level higher increased by about 35.4% for every one-day increase in the days to surgery. The conclusions are consistent with our original analysis. It is generally thought that HO increases and to be more manifest during longer-term observation [19]. Our finding does not agree with this common belief, and suggests longer follow-up interval does not necessarily lead to increased severity of HO.

Potential confounders for our study included the use of NSAIDS and radiation therapy for reduction of risk of HO. However, review of medical record of all patients showed that no patients received any prophylactic or therapeutic NSAIDS or radiation during the study period, and thus our study was not affected by these factors. Another potential confounder is the mechanism of injury (high velocity injury vs. low velocity injury). However, in our study sample 148 of 151 patients had hip fractures following a mechanical fall (i.e. low velocity injury) making our sample mostly homogeneous. Three patients had hip fractures following “motor vehicle accidents” according to electronic medical record, but precise circumstance of injury (e.g. what type of accident, speed of collision, etc) was not fully described. One patient had class 2 HO and two patients had class 4 HO, but effects of a high velocity injury on HO severity need to be further evaluated with a larger sample size. Finally, we did not correlate for use of medication other than NSAIDS, severity of trauma or post-operative rehabilitation.

The etiologies for the association between time to surgery and increased severity of HO remain undetermined. Our analysis has ruled out some plausible risk factors but did not identify the actual causative factor, which needs to be explored in further studies.

## Conclusions

Our study showed class 3 or greater HO was associated with longer time interval between time of acute hip fracture and surgery (median 6 days) compared to all other groups (HO class 0–2), which had similar time interval between the fracture and surgery (median 2 days). While it is not always possible, every possible effort should be made to minimize the delay in surgery to reduce the degree of HO.

## Data Availability

The dataset supporting the conclusions of this article is proprietary to Stony Brook University Hospital and will not be shared, because the hospital restricts sharing of the raw data with concerned personnel only. For permission to access the data, contact Department of Radiology, Stony Brook Medicine, HSC 4–120, Stony Brook, NY 11794, USA.

## Abbreviations

HO: Heterotopic ossification
ORIF: Open reduction and internal fixation
THA: Total arthroplasty
